# Bridging the Gap: From Photoperception to the Transcription Control of Genes Related to the Production of Phenolic Compounds

**DOI:** 10.3390/ijms25137066

**Published:** 2024-06-27

**Authors:** Adriana Volná, Jiří Červeň, Jakub Nezval, Radomír Pech, Vladimír Špunda

**Affiliations:** 1 Department of Physics, University of Ostrava, 710 00 Ostrava, Czech Republic; adriana.volna@osu.cz (A.V.); jakub.nezval@osu.cz (J.N.); radomir.pech@osu.cz (R.P.); 2 Department of Biology and Ecology, University of Ostrava, 710 00 Ostrava, Czech Republic; jiri.cerven@osu.cz; 3 Global Change Research Institute, Czech Academy of Sciences, 603 00 Brno, Czech Republic

**Keywords:** photoreceptors, radiation, temperature, transcription factors

## Abstract

Phenolic compounds are a group of secondary metabolites responsible for several processes in plants—these compounds are involved in plant–environment interactions (attraction of pollinators, repelling of herbivores, or chemotaxis of microbiota in soil), but also have antioxidative properties and are capable of binding heavy metals or screening ultraviolet radiation. Therefore, the accumulation of these compounds has to be precisely driven, which is ensured on several levels, but the most important aspect seems to be the control of the gene expression. Such transcriptional control requires the presence and activity of transcription factors (TFs) that are driven based on the current requirements of the plant. Two environmental factors mainly affect the accumulation of phenolic compounds—light and temperature. Because it is known that light perception occurs via the specialized sensors (photoreceptors) we decided to combine the biophysical knowledge about light perception in plants with the molecular biology-based knowledge about the transcription control of specific genes to bridge the gap between them. Our review offers insights into the regulation of genes related to phenolic compound production, strengthens understanding of plant responses to environmental cues, and opens avenues for manipulation of the total content and profile of phenolic compounds with potential applications in horticulture and food production.

## 1. Introduction

Phenolic compounds (PheCs) are a group of secondary metabolites with antioxidative properties and UV screening function [1]. It was documented that several abiotic environmental factors lead to their increased accumulation—for example drought, decreased temperature, increased soil salinity, and exposure to heavy metals or UV radiation [2,3,4,5,6]. From these reasons, their involvement in antioxidative defense is assumed as well as their contribution to the increased stress tolerance. It is also assumed that their accumulation in the upper layers of the epidermis can greatly affect the total amount of UV radiation penetrating the deeper layers of mesophyll and therefore prevent excess radiation from reaching the lower cell layers of the leaf [7]. These facts suggest the irreplaceable role of PheCs in plant protection against abiotic environmental cues, although to date, direct proof of their contribution to antioxidative protection or data supporting their involvement in the processes leading to increased stress tolerance are scarce. Therefore, we decided to summarize the current knowledge about the transcription control of genes related to phenolic compound production and to explain how the transcription factors (TFs) driving the expression are related to light perception via the photoreceptors.

In addition to UV radiation, another crucial environmental factor driving (among others) the accumulation of PheCs is the irradiance and spectral composition of photosynthetically active radiation (PAR). In general, incident radiation is perceived in two possible ways—by the direct and indirect light perceptions. Indirect light sensing does not require any specialized molecules and is perceived via the multiple sensory mechanisms related to photosynthetic performance, and thus the availability of sugars [8,9,10,11,12], or increased accumulation of reactive oxygen species [13,14,15] and related alterations in the redox state of the cells. On the contrary, direct light perception is ensured via specialized photoreceptors (more details can be found in Section 2.1, Section 2.2, Section 2.3, Section 2.4 and Section 2.5). These proteins have a light-absorbing molecule (this can be pigment, flavin nucleotide, or a cluster of aromatic amino acids) that is responsible for the perception itself. In general, after the light absorption, the redox state of the chromophore is changed [16] which leads to alterations in the higher-order protein structures (depending on the specific photoreceptor it can be either monomerization, dimerization, or changes in the protein folding) which in turn affects the capability to interact with specific proteins or protein complexes (more details about direct light perception in plants can be found in Section 2).

**Figure 1:**
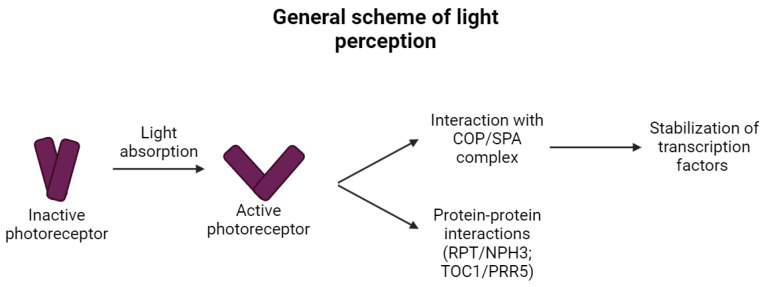
General scheme of direct light perception in plants (simplified). The absorption of light turns inactive photoreceptors into active states and causes conformational changes, which differ between the different types of photoreceptors (in some cases monomerization occurs, while in others dimerization, and other changes in higher-order structures are documented). Such changes significantly affect the protein–protein interactions that follow and are different for different photoreceptors. The vast majority of activated photoreceptors interact with the COP/SPA/E3 ubiquitin ligase complex and therefore lead to the stabilization of transcription factors driving the gene expression of target genes. The remaining photoreceptors (zeitlupes and phototropins) interact with other proteins and do not contribute to the COP/SPA signaling.

In general, after the light-induced activation of the photoreceptors (Figure 1), the interaction with the COP1/SPA (CONSTITUTIVE PHOTOMORPHOGENIC 1 and SUPPRESSOR OF PHYTOCHROME A-105) complex (more details can be found in Section 4) follows (with the exception of zeitlupes and phototropins). This large protein complex can induce the ubiquitination and proteolysis of several dozens of substrates including the transcription factors [17] affecting the expression of genes related to the production of phenolic compounds. Therefore, the COP1/SPA protein complex integrates the vast majority of the light sensing from the various photoreceptors [17] and coordinates the expression of target genes via balancing the availability of transcription factors (TF, especially of HY5 TF, which is involved in the induction of the transcription in genes related to the production of PheCs). More details about the structure, function, and interactome of the COP1/SPA complex can be found in Section 4.

The genes responsible for producing phenolic compounds can undergo regulation through either a single relatively specific transcription factor [18] which directly interacts with a particular region in the gene’s promoter, or they can form a specialized complex composed of several such proteins that collectively regulate the same gene [19]. Depending on the position in the biosynthetic pathway, we can distinguish the early and late genes related to the phenolic compounds production depending on the position in the biosynthetic pathway. Early genes have a rather simple regulatory mechanism via the single transcription factor (no complex is needed), which requires the presence of a specific sequence in the promoter region. On the contrary, the late genes related to the production of PheCs have a more complex regulatory control, which is ensured via the MBW complex consisting of three main components (M—MYB proteins, B—bHLH proteins, and W—WD40 repeat proteins). Therefore, for control of these “late genes” of PheCs biosynthesis, all three components of the MBW complex are necessary [19]. Details about the MBW’s complex structure, function, and regulation can be found in Section 5.6.

The final part of the review (Section 7. Future Perspectives) is dedicated to the summarization of the presented knowledge and highlighting possible applications in the horticulture and indoor cultivation of important agronomical plants and crops, which might lead to the production of more resilient plants with adjusted content of protective metabolites without the adverse effects of the imbalanced light conditions on plant production [20,21] and morphology. To reach these aims we have to elucidate the mechanisms and regulation of light signaling and their crosstalks with sensing of other environmental cues.

## 2. Direct Light Sensing

**Figure 2:**
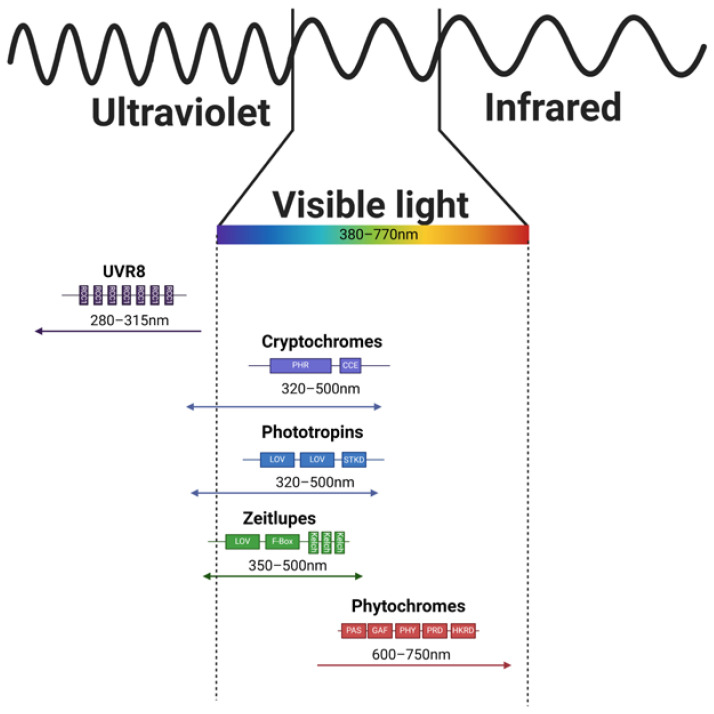
Domain architecture and absorption ranges of the photoreceptors. This scheme represents each group of known plant photoreceptors with highlighted absorption ranges, domain architecture, and the corresponding part of the spectra these photoreceptors play a role in (adopted and modified based on publications [22,23,24,25]).

**Figure 3:**
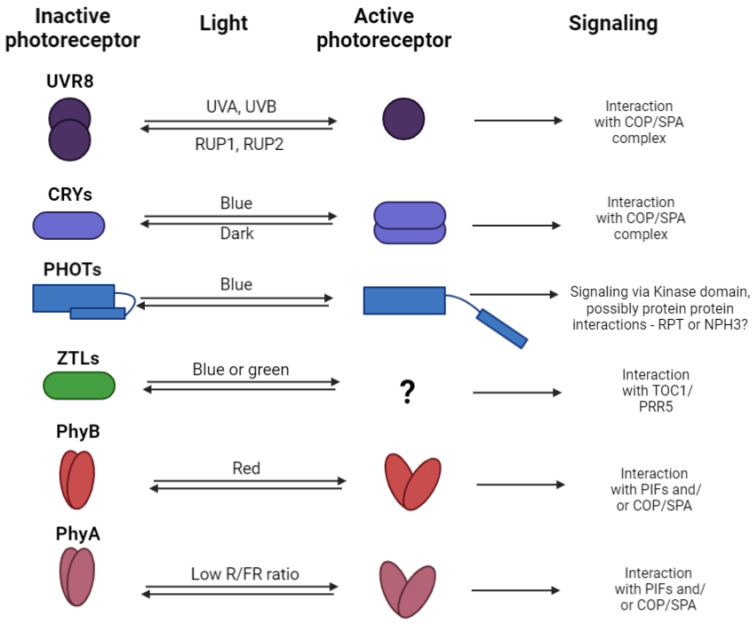
The mechanism of the photoreceptors sensing, signaling, and regeneration. For each plant photoreceptor, the basic mechanism of the photo sensing is indicated (it can be either monomerization, dimerization, or conformational changes), together with the light region that induced the active state of photoreceptors, and the signaling pathway where the light signal is further propagated (the related literature is cited in the text accompanying this scheme).

As mentioned above (Section 1), direct light sensing is ensured by specialized proteins called photoreceptors. These proteins vary in the domain architecture, structure, bound chromophore, and absorption properties (as depicted in Figure 2) and some of them even in the interactions after the light-induced activation (as shown in Figure 3).

### 2.1. Ultraviolet-B Receptor

The specific group of photoreceptors are UVR8s. The absorption range is from 280 to 315 nm [26] and differs for the monomeric and dimeric state [27], therefore these photoreceptors are able to absorb UV-A and also UV-B radiation.

The specificity of UVR8 lies in the fact that it does not contain a nonprotein chromophore, but instead, it has several RCC1 domains resulting in a beta-propeller structure with aromatic amino acids (tryptophans) situated at the propeller’s core. Tryptophans, being aromatic amino acids, possess the capability to directly absorb light and transmit excitation energy. Within the UVR8 protein, these amino acids aggregate into three tryptophan clusters, with six tryptophans forming the distal cluster, three in the proximal cluster, and four in the central cluster, effectively fulfilling the role typically played by nonprotein ligands (such as FAD or FMN) in other photoreceptors. In Arabidopsis, the transport of excitation energy within UVR8 is facilitated through resonance energy transfer [28], starting from the distal tryptophan cluster (W39, W92, W144, W196, W300, W352), then passing through the proximal cluster (W198, W250, W302), and finally reaching the central cluster (W233, W285, W337, W94). Although the ultimate destiny of the excitation energy remains unknown, it is established that conformational change occurs within the entire UVR8 structure, leading to UVR8 monomerization and consequent alterations in its potential interactions with other proteins.

Inactive UVR8 forms dimers, but once the UV radiation photon is absorbed, the dissociation occurs and UVR8 forms an active monomer. Active UVR8 monomer binds the COP1/SPA/E3 ubiquitin ligase complex and induces COP1/SPA dissociation from the enzymatic complex (CUL4/DDB1/E3 ubiquitin ligase; Figure 3). Active complex UVR8/COP1/SPA then migrates to the nucleus. Here, the UVR8/COP1/SPA complex directly interacts with the HY5 transcription factor and therefore increases its stability. In addition, HY5s binding to its own promoter [29] can be induced.

The equilibrium between the active and inactive UVR8 photoreceptor [30] is maintained via the interaction with the RUP proteins (specifically the RUP1 and RUP 2 sharing 63% of their sequence [31]), which physically interact with the monomeric UVR8 protein and facilitate its return to the ground dimeric state [32].

### 2.2. Cryptochromes 

Direct light sensing occurs through photoreceptors that operate within specific spectral intervals of solar radiation (Figure 1). Blue light is detected by a diverse group of photoreceptors, including cryptochromes (CRYs), phototropins (PHOTs), and zeitlupes (ZTLs). CRYs, which are relatively short proteins (over 480 amino acids in *Hordeum vulgare*), bind flavin adenine dinucleotide, enabling light absorption [33] in the range of 320–500 nm [34]. CRY1 and CRY2 are distinguished within the CRYs, and their signaling can modulate the expression of 10–20% of *Arabidopsis thaliana* coding genes [35]. In monocot plants, CRY1 is encoded by two different genes, designated CRY1a and CRY1b, while CRY2 is encoded independently, differing from CRY1 in gene sequence length, exon–intron structure, presence or absence of 5′UTR, and amino acid composition of the resulting protein [36]. Structurally, they contain PHR (Photolyase Homologous Region) and CCE (CRY C-terminal Extension) domains [36]. In addition to CRY1 and CRY2, *Arabidopsis thaliana’s* genome also contains CRY3 [37]. Unlike CRY1 and CRY2, CRY3 exhibits a distinct structure characterized by the absence of a C-terminal extension necessary for COP/SPA complex interaction [37]. This structural variance translates into functional differences as well. It appears that CRY3 diverges from the typical photoreceptor role in plants and instead functions as a DNA photolyase [37]. Homologs of CRY3 were also identified in other plant species—for example in tomato and rice [37].

The general model of CRYs sensing involves blue light absorption by incorporated FAD, leading to conformational changes that form CRY homodimers. The active dimer is phosphorylated and interacts with the COP1/SPA (Figure 3) E3 ubiquitin ligase complex, which leads to the initiation of gene expression [38]. Additionally, the transition of CRYs from monomers to dimers can be repressed by interaction with BIC1 and BIC2 proteins (BLUE-LIGHT INHIBITOR OF CRYPTOCHROMES 1, and BLUE-LIGHT INHIBITOR OF CRYPTOCHROMES 2), providing additional signaling control. In addition, recent knowledge suggests the potential for CRYs to form tetramers [39,40,41].

### 2.3. Phototropines 

The second group of blue light photoreceptors, PHOTs, are approximately 900 amino acids long in *Arabidopsis thaliana* and possess two LOV (Light-oxygen-voltage-sensing domain) domains, a serine/threonine kinase domain, and a Jα helix [42]. Light absorption by their chromophores, FMNs [43], induces conformational changes, activating the kinase domain [43,44,45,46]. Although the PHOT signaling pathway is not fully elucidated, proteins such as NPH3 [47] and RPT2 [48,49] have been identified to interact with PHOT1(Figure 3), playing roles in photoreceptor complex constitution, phototropism, and stomata opening.

### 2.4. Zeitlupes 

Another set of photoreceptors capable of light perception is represented by adagio proteins. These proteins are known for sensing in the blue/green light regions, with an absorption range from 350 to 500 nm [50], covering UVA, blue, cyan, and green spectral components. ZTLs feature LOV, F-box, and kelch domains, with the LOV domain, containing flavin (FAD, FMN, or riboflavin [51]) bound to the LOV protein via cysteine residues. Together with FKF1 (Flavin-kelch-Fbox-1) and LKP (LOV kelch protein 1), ZTLs primarily function in sensing photoperiod duration and flowering time [50]. ZTLs and FKF1 can form heterodimers with the GI protein, induced by blue light. Decreasing light dose [50], particularly its blue component, during light/dark transitions releases ZTL from the GI (GIGANTEA) complex, enabling interaction with TOC1 (Timing Of Cab expression 1; transcription repressor) for degradation during the dark period, thereby influencing the transcription of target genes. Similarly, decreasing light dose leads to the release of FKF1 from the GI heterodimer, allowing interaction with CDF (Cycling Dof Factor) to trigger the transcription of related genes [50,52].

### 2.5. Phytochromes

The exclusive photoreceptors responsible for perceiving red and far-red light are phytochromes (PHYs). Phytochromes are approximately 124 kDa proteins that form homodimers and contain covalently bound pigments, specifically phytochromobilin [53]. Their tertiary structure comprises N-terminal extension, PAS (per-ARNT-sim), GAF (cGMP phosphodiesterase/adenylyl cyclase/FhlA domain), PHY (phytochrome specific domain), PRD (PAS-related domain), and HKRD (histidine kinase-related domain) [54]. The phylogenetic analysis allows the distinction of phytochrome sub-family members, which appear to be genus specific, resulting in varying numbers of members among plant species. For instance, *Arabidopsis thaliana* exhibits PhyA-PhyE, while spring barley displays PhyA-PhyC [42]. However, studies indicate that PhyA [55] and PhyB [56,57] are the most crucial for environmental sensing, while the roles of other PHY family members remain to be fully elucidated. The precise mechanism of Phy-related signaling will be detailed below.

**Figure 4:**
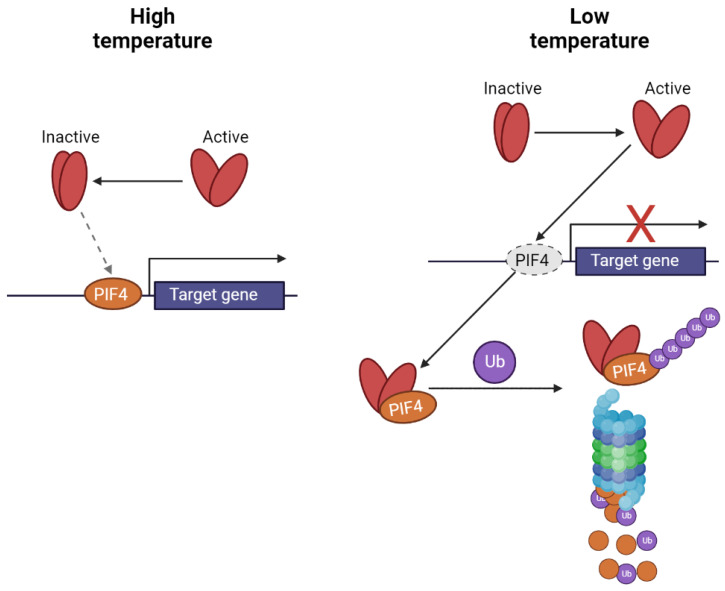
Mechanism of the transcription control of target genes by PhyB interaction with PIFs (scheme was adopted from [60] and modified).

Phytochromes exist in two distinct states: inactive (Pr) and active (Pfr), whose transitions are triggered by red and far-red radiation, respectively, as depicted in Figure 4 and their respective ratios. While red light activates PhyB, far-red light deactivates it (a process that can be induced by increased temperature). These transitions involve conformational changes that result in shifts of absorption spectra and alter the potential for interaction with other proteins. When in the active state, phytochrome can interact with phytochrome-interacting factors (PIFs—PIF4 and 7), which are responsible for positively controlling gene expression as transcriptional activators. This interaction leads to ubiquitination and subsequent degradation by the 26S proteasome, thereby reducing gene expression. In contrast, when phytochromes are inactivated (due to increased temperature or exposure to far-red light), they are unable to interact with PIFs, allowing for the initiation of transcription of target genes [56,58].

PIFs target genes that contain G-boxes in their promoters, such as genes related to PheCs biosynthesis which feature this sequence motif, or G/PBE boxes. PIFs can form homodimers or heterodimers with different PIFs or completely different proteins [59]. An example of this is their interaction with HY5 ([59] Protein LONG HYPOCOTYL 5), a participant in light-related signaling.

In addition to their direct interaction with activated phytochromes, the transcription of PIFs (such as PIF4) and the translation of PIF7 are finely regulated by alternative mechanisms. For instance, under lower temperatures, the expression of PIF4 is inhibited by the ELF3 (EARLY FLOWERING 3) protein. However, as temperatures rise, ELF3 undergoes phase separation, leading to the initiation of transcription of target genes. Similarly, a hairpin structure localized in the 5′-UTR of PIF7 mRNA undergoes a temperature-induced change in structure, directly correlating with increased synthesis of PIF7 proteins [61].

## 3. Photoreceptors and Temperature

Furthermore, photoreceptors involved in UV and blue/green light sensing, including PHOTs, CRYs, ZTLs, and UVR8 [62], may also participate in thermosensing processes. For example, in *Marchantia polymorpha*, PHOTs exhibited prolonged activity under decreased temperatures [63], while in *Arabidopsis thaliana*, a greater population of active CRYs was observed at lower temperatures compared to moderate ones [64]. The activity of UVR8 photoreceptors also appears to be influenced by temperature, with a reduced proportion of inactive UVR8 dimers observed at lower temperatures [30], indicating increased monomerization. Additionally, the number of active UVR8 monomers can be modulated by RUP (REPRESSOR OF UV-B PHOTOMORPHOGENESIS1 and REPRESSOR OF UV-B PHOTOMORPHOGENESIS2) proteins [30]. ZTL inactivation progresses with increasing temperatures, yet ZTL proteins are believed to be involved in heat stress tolerance, as evidenced by studies with ZTL-deficient and ZTL-overexpressing mutant lines of *Arabidopsis thaliana* [65]. Thus, plant photoreceptors not only play a primary role in photosensory functions but also share a role in thermosensing, regulated by temperature-induced changes in their active state duration. Lower temperatures prolong the duration of the active state, whereas higher temperatures decrease it. While an increasing number of publications support this assumption, direct experiments are required to confirm or refute this hypothesis. Additionally, plants possess numerous other thermosensing mechanisms [66] beyond the scope of this review.

## 4. Constitutive Photomorphogenesis Protein 1/Protein SPA1-Related Protein Complex

The majority of activated photoreceptors, excluding zeitlupes and phototropins, interact with the COP1/SPA protein complex (see Figure 3). This complex comprises the COP1 protein, which exists as a single copy gene in *Arabidopsis thaliana*, and one of four SPA proteins. Research has shown that COP1/SPA is part of a larger complex alongside CUL4 (Cullin-4), DDB1 (DNA Damage-Binding Protein 1), and RBX (RING-Box Protein) [17].

In *Arabidopsis thaliana*, this enzymatic complex plays a role in degrading numerous proteins, including transcription factors (TFs) such as BIT1, HY5, HYH, PIF1, PIF5, PIF8, and PIL1, as well as photoreceptors (CRYs, PHYs) [17] involved in various processes like photomorphogenesis, light perception, anthocyanin biosynthesis, and phytohormone signaling. Typically, in darkness, different substrates, primarily TFs, undergo ubiquitination, leading to the suppression of target genes. However, upon light activation of photoreceptors, the activity of the COP1/SPA complex is inhibited, resulting in reduced substrate degradation and increased TF population, thereby activating downstream genes. Although the COP1/SPA complex generally plays an inhibitory role by inducing substrate degradation, it exhibits unique interactions. For instance, when interacting with activated UVR8, it stabilizes the HY5 transcription factor. Additionally, in association with PHYs, it facilitates the degradation of PIFs, transcriptional repressors.

## 5. Transcription Factors Involved in PheCs Biosynthesis Control

PheCs biosynthetic pathway forms an important branch of higher plant secondary metabolism. PheCs content is effectively regulated by several environmental cues and their co-action (such as PAR, UV, LT, low N, high CO_2_, etc.) and they are often studied for their possible contribution to stress-related responses and enhancement of plant tolerance. Below, we intend to describe in detail how the transcriptional control of genes is directly involved in the production of such universal/multipurpose substances. TFs responsible for the coordination of the gene expression contain one of the following motifs: bHLH, bZIP, HTH, or WRKY, and are listed in Table 1.

### 5.1. Proteins Containing Basic Helix–Loop–Helix Motifs (So-Called bHLH)

This diverse group of proteins contains (as suggested by the acronym bHLH) a basic helix–loop–helix motif and comprises 26 protein subfamilies [67], and all of them are responsible for direct interaction with the DNA and transcription control. These proteins are present in almost all plant species and in some of them, more than 100 different bHLH proteins were documented. The estimated origin of this group of proteins dates back 440 million years ago to the first land plants [67]. They are involved in the various processes under both optimum [68] and stress conditions [69].

An important protein containing a bHLH structural motif is TT8 (TRANSPARENT TESTA 8; also known as AtbHLH42, bHLH42, or Transcription factor EN32) [42]. According to the literature, this protein is a 518 amino acids long transcription activator, when associated with MYB75 (PAP1) or MYB90 (PAP2) [42]. In addition, this protein controls the expression of the DFR (DIHYDROFLAVONOL 4-REDUCTASE; Table 1) and BAN (BANYULS) genes [70] in *Arabidopsis thaliana* siliques. DFR gene codes for the DFR protein which catalyzes the formation of leucopelargonidin from dihydrokaempferol or the formation of leucocyanidin from dihydroquercetin [70]. BANYULS gene codes for a DFR-like protein [71] which is a negative regulator of anthocyanin biosynthesis. Plants with knocked-out BAN displayed a higher accumulation of anthocyanins in the seed coat. In addition, it was even proposed that this gene codes for leucoanthocyanin reductase (LAR), but no direct evidence was reported. On the contrary, it was documented that BAN genes of *Medicago truncatula* and *Arabidopsis thaliana* encode for anthocyanidin reductase catalyzing the formation of 2,3-cis-flavan-3-ols from anthocyanins and therefore the reduction in coloration [72].

### 5.2. Basic ZIPper-Containing Proteins (bZIP)

The second group of the TFs related to the PheCs production are basic (leucine) zipper (bZIP)-containing proteins (Table 1). Their common ancestor (the divergence point varies depending on the plant species; monocots and eudicots are estimated 140–150 million years ago) probably had four bZIP genes, and those proteins were probably involved in the oxidative stress responses and also in the responses to light [73]. This assumption is supported also by current literature—where bZIP family members are referred to as key players in the transcription control of the genes triggered in the response to light and photomorphogenesis. Moreover, this diverse group of TFs is involved also in responses to other abiotic stresses [74], as well as in the responses to pathogens [75].

The most important member of this protein family is AtZIP56 (*Arabidopsis thaliana* basic leucine zipper 56) also known as HY5 (ELONGATED HYPOCOTYL 5). This TF interacts directly with the G-box motif (-GCCACGTGC/GC-; but interactions with E-box, GATA-box, Z-box, C-box, and other hybrid boxes of the DNA, including promoters related to the PheCs production, are documented as well [76]). Due to the interaction with the COP/SPA protein complex, which is involved in the signaling of the environmental cues (both light intensity and spectral quality, as well as temperature), HY5 is designated as an “integrator of light and temperature signals” [77]. In *Arabidopsis thaliana*, HY5 is a 168 amino acids long protein containing disordered bZIP, leucine zipper, basic motif, and an interaction site for COP1 (-ESDEEIRRVPEF-; [42]). In the dark, HY5 is ubiquitinated after the interaction with the COP1 and degraded at the 26S proteasome complex [78]. On the contrary, in the light COP1 is inhibited, and therefore the proteins HY5/HYH/CAM7 (HY5—ELONGATED HYPOCOTYL 5, HYH—HY5 HOMOLOG, CAM7—Calmodulin-7) can interact with the HY5 promoter and induce HY5s transcription. This results in the temporary accumulation of HY5 proteins which can trigger the expression of the genes with the G box [78]-containing promoters, including those related to the PheCs production.

The second and equally important member of the bZIP family is HYH (HY5 HOMOLOG). In *Arabidopsis thaliana* it is 149 amino acids long protein with the disordered, bZIP, basic motif, leucine zipper regions, and interaction site for COP1 [42]. HY5 and HYH share an identity of 49% (73 out of 149 amino acid residues), and in the DNA-binding region, the homology in the amino acid sequence steeply increases to 87.5% (21 out of 24 amino acid residues) [79]. Similarly to HY5, also HYH is involved in the regulation of the expression of genes related to the biosynthesis of PheCs. It was documented that HYH directly interacts with the COP1’s WD-40 domain and the main consequence is the degradation of the HYH protein. In addition, both proteins (HY5 and HYH) are involved in the regulation of the transcription in the same pathway—biosynthesis of anthocyanins under the low temperature in the *Arabidopsis thaliana* [80].

### 5.3. Transcription Factors Containing Helix–Turn–Helix Motifs (HTH TFs)

The third group of the TFs is helix–turn–helix motif-containing proteins (HTHs). MYB proteins (MYBs; the acronym is derived from Avian myeloblastosis virus, where the first protein containing such structure was reported), for example, belong to this diverse group of proteins. MYBs have a conserved DNA-binding domain that consists of amino acid repeats (R meaning one repeat in the structure descriptions below) which can be imperfect and are approximately 50 amino acids long. Each of the repeats forms α-helices, but the most important are the second and third helices of each repeat—those directly recognize (the third one), interact (the second one) with the DNA, and form HLH structure [81]. In *Arabidopsis thaliana,* 197 genes coding for MYB TFs were identified in all 5 chromosomes which vary in the number of the repeats and therefore form 4 subfamilies [82]: MYB-R1R2R3, MYB-R2R3, MYB-related genes, and atypical MYB genes.

TT2 (Protein TRANSPARENT TESTA2, also known as AtMYB123) is a 258 amino acids long transcription factor. When associated with bHLH2/EGL3/MYC146 (basic Helix Loop Helix protein 2, also known as Protein ENHANCER OF GLABRA 3, or Transcription factor EN 30), bHLH12/EN52/MYC1 (basic Helix Loop Helix protein 12, also known as Transcription factor EN 58, or Myelocytomatosis protein 1), or bHLH42/tt8 (basic Helix Loop Helix protein 42, also known as TRANSPARENT TESTA8) work as transcription activator involved in the control of late anthocyanin genes (Table 1; [83]).

In *Arabidopsis thaliana*, PAP1 (Probable plastid-lipid-associated protein 1, also known as FBN1a, FIB1a, or PGL35) is 318 amino acids long protein involved in the transcription control (genes controlled by the PAP1 are mentioned below). Expression of this protein stimulates the jasmonic acid biosynthesis and it was documented that increased accumulation of PAP1 is linked with the increased tolerance of the PSII against photoinhibition in Arabidopsis [84]. On top of that, the PAP1 protein is a key player in the PheCs regulation for example in *Arabidopsis thaliana* [85], *Salvia miltiorrhiza* [86], *Brassica napus* [87], and *Nicotiana tabacum* [88].

PAP2 (also known as FBN1b or FIB1b) is 310 amino acid residues long transcription factor in *Arabidopsis thaliana*. Similarly to PAP1, PAP2 is involved in stress responses and its expression also stimulates jasmonic acid biosynthesis [89]. In addition, PAP2 in *Arabidopsis thaliana* is involved in the protection of PS II against photooxidative stress (induced by high light in combination with cold). According to the Plant Transcription Factor Database (Table 1), PAP2 stimulates the expression of TTG2 in *Arabidopsis thaliana*. Therefore, both proteins (PAP1 and PAP2) are connected with growth regulation and PSII protection, but also with anthocyanin accumulation under stress conditions [89].

According to the current knowledge, other members of the MYB family are equally important. An example of these members is the trio MYB11, MYB12, and MYB111, which are involved in the control of genes involved in PheCs biosynthesis. All three proteins have the same type of the MYB domain—R2R3, have a similar length (MYB11—343 amino acids, MYB12—371 amino acids, MYB111—342 amino acids), and are designated as flavonol-specific TFs because all of them positively regulate the transcription of the *CHS*, *CHI*, *F3H*, and *FLS1* genes in *Arabidopsis thaliana*.

### 5.4. WRKY Proteins

This group of TFs is also involved in very diverse processes related to various types of abiotic stresses [90]. The characteristic of those TFs is that all members of the WRKY family have WRKYGQ repetitions near the N-terminus and can directly interact with W-box ((C/T) TGAC (T/C))-containing promoters [90]. In *Arabidopsis thaliana*, there were identified 74 genes coding for the WRKY TFs [90]. W-box motif in the promoter region is present mainly in genes involved in the biosynthesis of lignin [91], flavonols, or tannins [92]. WRKY TFs therefore contribute to the control of the secondary metabolism. For example, in the *Arabidopsis thaliana*, WRKY23 probably controls the expression of *TT4*, *TT5*, *TT6*, and *TT7* genes [93] (TRANSPARENT TESTA proteins 4–7; the genes coding them are also known as *CHS*, Chalcone-flavanone isomerase, Naringenin,2-oxoglutarate 3-dioxygenase, and Flavonoid 3′-monooxygenase) [42]. The WRKYGQ repeat tract is responsible for the interaction with the DNA, which directly interacts with the W-box sequence in the major groove as was documented in *Solanum lycopersicum* (WRKY3 and WRKY4) [94].

Several subclasses of the WRKY proteins were described based on their structure—I, II, and III. Group I WRKY proteins have two WRKY domains, group II proteins have one WRKY domain and one zinc finger (Cys2-His2) motif, and group three have one WRKY domain and one zinc finger (but different from the group II—Cys2-His/Cys Cys2-His2 zinc-finger motif) [95]. In addition to the numeric description of the domain architecture, other higher-order structure features can be indicated by the lowercase letters a–e. The wide structural diversity of the WRKY proteins therefore enables them to interact with each other and further increase their regulatory potential. For example, in the *Arabidopsis thaliana*, members of the IIb group can interact with each other (AtWRKY6/AtWRKY42), but also with the IIa group (AtWRKY36/AtWRKY40). Similarly, the WRKY proteins belonging to the IIa group can both interact with each other or with the IIb group members (AtWRKY36/AtWRKY60). Lastly, the members of group III can interact with each other (AtWRKY30/AtWRKY53), but also with the IIa group members [95] (AtWRKY40/AtWRKY38). The limited regulatory potential of the IId members is quite interesting, whose exclusive interaction with the IIa group (OsWRKY71/OsWRKY51) was documented in *Oryza sativa*. In addition to this (WRKY-WRKY protein interactions), the interactions with other proteins were documented. For example with the VQ motif-containing proteins (HAIKU1, MAPK4, MKS1, or even with the chromatin remodeling proteins (HDA19) [96].

An interesting member of the WRKY TFs family involved in light-related signaling control is WRKY36, which is the transcriptional inhibitor of the *HY5* gene coding for the HY5 protein. It was documented that UVR8 can directly interact with the WRKY36 in *Arabidopsis thaliana* and also that UVB exposure increases the amount of UVR8/WRKY36 dimers [97]. In combination with the fact that UVB leads to increased UVR8 accumulation in the nucleus, authors proposed [97] that those active UVR8 monomers (formed after the UVB exposure) migrate to the nucleus and pull the WRKY36 transcription inhibitor from the promoter of the *HY5* gene, and thus lead to the increased transcription of the central light and temperature integrator (HY5) which controls both other TFs (e.g., MYB12) as well as PheCs-related genes.

### 5.5. WD-40

WD-40 proteins are characterized by a WD-40 domain containing multiple repeats [98], where each repeat contains 44–60 amino acids [99]. Each unit contains dipeptides (either glycine-histidine (GH; in the proximity of the N-termini) or tryptophan-asparagine (WD; in the proximity of the C-termini)) and can be folded into the four-stranded antiparallel beta-sheet, which is one segment of the beta-propeller structure (a similar structure to the UVR8 photoreceptor in the monomeric state; [99]). Each WD-40 protein has at least four such segments [98].

In *Arabidopsis thaliana*, 269 WD-40 proteins were identified and can be distinguished in approximately 113 subfamilies which have significant homology with their human or fruit fly counterparts suggesting their importance [99].

For example, the COP1 (CONSTITUTIVE PHOTOMORPHOGENIC 1) protein is a member of the WD-40 family having seven WD repeats in Arabidopsis [98]. This particular protein is involved in the light signaling and control of photomorphogenesis via the direct repression of the transcription activators [98]. Recently, it was also documented that it stabilizes the transcription repressors (PIFs, EIN3, or EIL1; [100]).

Another example of the WD-40 protein can be SPA proteins [101]. In *Arabidopsis thaliana*, four SPA (SPA1, SPA2, SPA3, SPA4) proteins were identified and each one of them can interact with the COP1 [101].

### 5.6. MYB-bHLH-WD-40 Complex

For a late group of PheCs-related genes, transcription is controlled at multiple levels. In addition to the direct interaction of the promoters with the various TFs (for example TT2), the MBW complex (consisting of the MYB-bHLH-WD-40 proteins) also contributes to the coordination of the gene expression. In addition, *Arabidopsis thaliana* has two different MBW complexes—the first one for the control of the anthocyanin (PAPs-EGL3/GL3/TT8-TTG1) (Lloyd et al., 2017) and the second one for the control of proanthocyanidin (TT2/TT8/TTG1) biosynthetic genes [102] in the plant body (anthocyanins) and seeds (proanthocyanidins)—which further highlight the importance of MBW complex. In this complex (MBW) the PAP1 and TT8 are responsible for the DNA binding, and consequent regulation of transcriptional activity, while the TTG1 is necessary for the MBW complex activity; and it is assumed that it also prevents the promoter interaction with the transcriptional inhibitors [77].

The MBW complex itself positively regulates transcription of the PheCs-related genes, but interactions with other proteins are documented and result in the alteration (usually the decrease) of the late genes expression. For example, MYBL2 (MYB-like 2 protein) probably competes with the R2R3 MYBs at the MBW complex and interacts with the bHLH proteins which leads to decreased anthocyanin production [103]. Similarly, the SPL9 (SQUAMOSA PROMOTER BINDING PROTEIN-LIKE 9) protein directly competes with the TT8 protein for interaction in the anthocyanin-related MBW complex. If the SPL9 is incorporated, the reduced transcription of the target genes related to the anthocyanin biosynthesis is observed (DFR, BAN, etc.) in *Arabidopsis thaliana* [103], and therefore (FLS and DFR compete for substrates) the flavonol biosynthesis [103] is increased. In addition, expression of the SPL9 is controlled at the posttranscriptional level by the miR156 and miR157 via PTGS [103].

## 6. Main Transcription Regulators Coordinating Expression of Genes Related to the Production of the Phenolic Compounds

To further summarize available knowledge focused on the TFs driving the expression of genes related to the production of phenolic compounds, we selected 13 crucial TFs based on different structural motifs mentioned above in the text. We browsed the corresponding information in the PlantTFDB database [104] for *Arabidopsis thaliana*. These selected TFs are summarized in Table 1, including the information about their structure and target genes these regulate (positively and if any, negatively as well).

Firstly, the TT8 TF (having the bHLH structural motif) according to the database positively affects the expression of the genes related to the phenolic compounds production (BAN, and DFR), putative methylesterases (MES4, and MES6), WRKY44 TF (TTG2), bZIP TF (GL2), MYB-like 2 and its own expression. From the data in the database, so far, no gene was documented to be repressed by the TT8.

Although the vast majority of the reported studies documented the stimulative effects of the HY5 on the target genes, according to the PlantTFDB database (Table 1), three target genes are repressed, by the HY5 TF—NPF6.3 (gene coding for NRT1 Protein—that is also known as Nitrate transporter 1.1), FHY1 (gene coding for FAR-RED ELONGATED HYPOCOTYL 1 Protein, involved in the FR/R responses), and FHL (gene coding for the FAR-RED-ELONGATED HYPOCOTYL 1-LIKE Protein). On the other hand, HY5 positively regulates the expression of 19 genes related to various processes—from those related to the production of phenolic compounds (MYB12, CHS, DFR, UGT84A1, LDOX), via those related to the production of chlorophyll and photosynthesis (HEMA1, ELIP1, CAB2, CAB1, PSBD, RBCS1A), those related to the phytohormones (IAA7, IAA14, ABI5, HB-8), and those related to the nitrogen and phosphorus metabolism (NIA2, PHR1), or light responses (ELF4, LZF1).

HY-5like TFs are similar in function, namely HYH1, HYH2, HYH3, and HYH4 targeting genes that activate (PHR1, NIA2, ELIP1, PEX11B, CHS) and repress (NPF6.3) as well as TT2 TF, similar to TT8 regulating the expression of genes related to phenolic compound production (ANS, DFR, BAN) and other TFs (TT8, TTG2, GL2), and its own as well (TT2).

**Table 1:** Summary of selected plant transcription factors of *Arabidopsis thaliana* related to the control of the genes involved in PheCs biosynthesis (data were extracted from the PlantTFDB database [104]; question marks represent no available data in the source database dealing with the activated or repressed target genes). Dash represents no detected repressed genes, while question marks indicate no available data in the database.

**TF**	**TT8**	HY5	HYH_1	HYH_2	HYH_3	HYH_4	TT2	PAP1	MYB11	MYB12	MYB111	WRKY23	WRKY36
**Motif**	bHLH	bZIP	bZIP	bZIP	bZIP	bZIP	HTH	HTH	HTH	HTH	HTH	WRKY	WRKY
**ID**	AT4G09820	AT5G11260	AT3G17609.1	AT3G17609.2	AT3G17609.3	AT3G17609.4	AT5G35550.1	AT1G56650.1	AT3G62610.1	AT5G49330.1	AT5G49330.1	AT2G47260.1	AT1G69810.1
**Target genes activated**	*BAN*, *TT8*, *MYBL2*, *DFR*, *GL2*, *MES6*, *MES4*, *TTG2*	*RBCS1A*, *ELIP1*, *PHR1*, *LZF1*, *IAA7*, *CHS*, *CAB2*, *ABI5*, *IAA14*, *DFR*, *CAB1*, *UGT84A1*, *PSBD*, *NIA2*, *ELF4*, *LDOX*, *HEMA1*, *MYB12*, *HB-8*	*PHR1*, *NIA2*, *ELIP1*, *PEX11B*, *CHS*	*PHR1*, *NIA2*, *ELIP1*, *PEX11B*, *CHS*	*PHR1*, *NIA2*, *ELIP1*, *PEX11B*, *CHS*	*PHR1*, *NIA2*, *ELIP1*, *PEX11B*, *CHS*	*ANS*, *TT8*, *TT2*, *TTG2*, *GL2*, *DFR*, *BAN*	*CHS*, *CHI*, *DFR*, *MYB3*, *TT8*, *UF3GT*, *PAP2*, *A5GT*, *UGT78D2*, *5MAT*, *MBD2.2*, *GST*	*CHS*, *CHI*, *F3H*, *FLS1*	*CHS*, *CHI*, *F3H*, *FLS1*	*CHS*, *CHI*, *F3H*, *FLS1*	???	???
**Target genes repressed**	-	*NPF6.3*, *FHY1*, *FHL*	*NPF6.3*	*NPF6.3*	*NPF6.3*	*NPF6.3*	-	*scpl10*	-	-	-	???	???

Although the PAP1 TF has been repeatedly documented to have a positive effect on the transcription of the target genes, according to the PlantTFDB database it inhibits transcription of the scpl10 gene coding for the Serine carboxypeptidase-like 10 protein that is involved in the sinapoylation of anthocyanins (adding of the acyl group to the anthocyanins consequently affecting the antioxidative properties, charge, and therefore probably the localization within the cell as well). On the other hand, PAP1 positively regulates the transcription of the genes related to the production of the phenolic compounds (CHS, CHI, DFR, UF3GT, A5GT, UGT78D2, 5MAT), but also other proteins and transcription regulators (GST, MBD2.2, MYB3, TT8).

The other three selected proteins with a HTH structural motif are MYB11, MYB12, and MYB112. All these three proteins do not (according to the database) repress any target gene, but positively affect the transcription of genes related to the production of flavonols (CHS, CHI, F3H, FLS). The last two selected proteins containing the WRKY structural motif are WRKY23 and WRKY36—although these TFs are reported in the literature as proteins affecting the expression of genes related to the production of phenolic compounds [95,97], so far, the PlantTFDB database does not have any records about their target genes—neither activated, nor repressed.

To conclude from above, TFs first regulate the target genes related to the phenolic compounds (but some of them are also other key enzymes—one example can be the HY5 positively regulating the expression of the genes related to the photosynthesis, biosynthesis of chlorophyll, and phytohormones), secondly, they can regulate each other’s expression (for example the TT2 TF positively regulates the expression of TT8 TF) and lastly, some of them have an autoregulatory function (for example, the TT8 TF positively regulates its own gene expression). In addition to this, some of these TFs also have an inhibitory role in the expression of some target genes (for example, the HY5 homologs with negative effects on the expression of NPF6.3 gene coding for the nitrate transporter). On the other hand, transcription control of these genes is not ensured exclusively by the TFs—so far, it has been shown, that alterations in the epigenetic modifications [105] or formation of local structures [106] have a considerable role as well.

An example can be the transcription regulation via the micro RNAs (miRNAs). These are relatively short (22–35 nucleotides long) RNA molecules complementary to “common” mRNAs (coding the final protein) able to either pause, or stop the expression of its target (via the post-transcriptional gene silencing, or RNA-induced methylation of the gene) [107]. Although it seems unlikely, miRNAs also contribute to the control of the production of phenolic compounds (e.g., miR156, miR858, or miR828). For example, miR858 [108] targets the MYB11, MYB12, and MYB111 TFs transcripts (with stimulative effects on the expression of genes coding for the enzymes of the phenylpropanoid pathway), but other members of the MYB R2R3 family are also putative targets of mir858. Similarly, the miR828 [109,110] targets the MYB TFs driving the expression of genes related to anthocyanin production via the degradation of MYB113, MYB82, and TAS4 (trans-acting small interfering RNA). This results in the negative regulation of MYB75 (PAP1) and MYB90 (PAP2)—here, a regulatory loop between PAP1 and TAS4 was also proposed (while PAP1 stimulates the accumulation of TAS4, the TAS4 has an inhibitory effect on PAP1, PAP2 and MYB113) [111].

**Figure 5:**
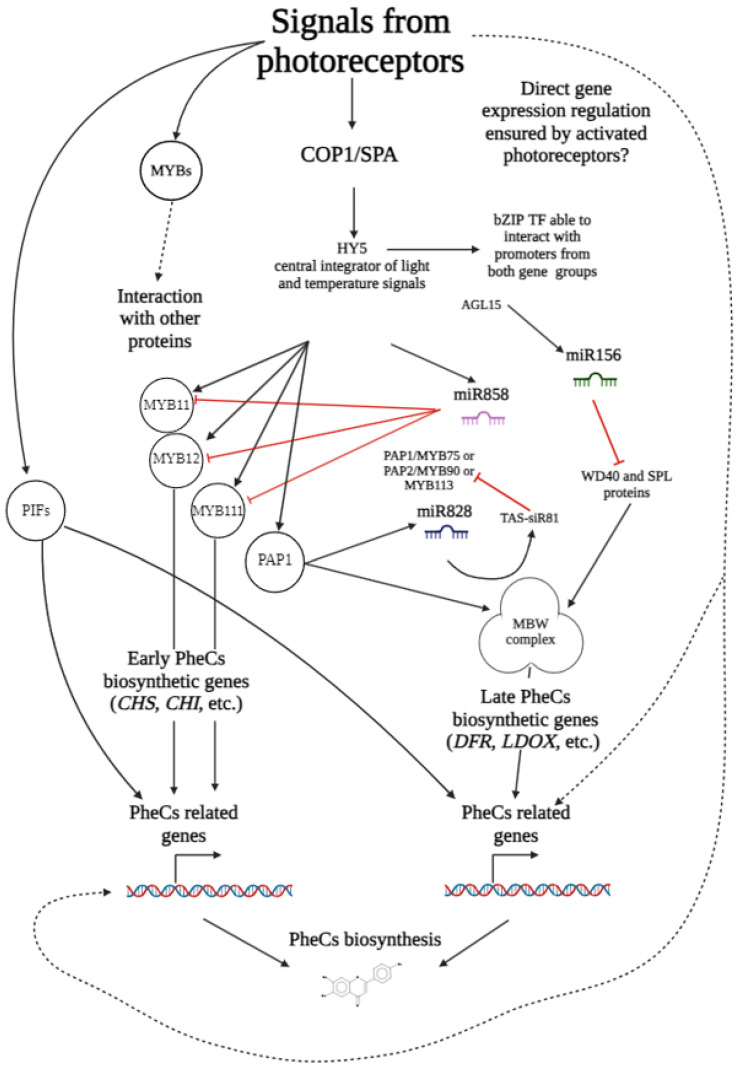
The simplified scheme of the transcriptional control of the genes related to the production of phenolic compounds (scheme was adopted from [112] and modified).

To better explain the transcriptional control of the genes related to the production of the phenolic compounds, we summarized the text from this section into a simplified scheme below (Figure 5).

## 7. Future Perspectives

In future studies, it would be beneficial to investigate the extent to which different photoreceptors drive the genes of interest. For instance, genes related to the production of phenolic compounds are known to be influenced by photoreceptors and their associated signaling cascades. While it is well documented that blue light and UV radiation can stimulate these genes, the specific contributions of various photoreceptors remain unclear. For example, cryptochromes are known to play a major role in blue light stimulation, but it is uncertain whether ZTLs or phototropins also contribute to this effect, and if so, to what extent. Additionally, how signals from multiple photoreceptors are integrated and prioritized, particularly in natural sunlight that activates more than one photoreceptor, needs further exploration. Understanding how the COP/SPA/Cul/DDB complex differentiates between signals from different photoreceptors and whether subsequent steps in the COP/SPA signaling cascade vary based on the originating photoreceptor is crucial.

Regarding nomenclature, establishing unified and widely accepted acronyms for key transcription factors would be highly beneficial. Currently, transcription factors involved in regulating phenolic compounds often have multiple aliases, complicating literature reviews and our understanding of light-induced responses. For example, PAP2 (Probable plastid-lipid-associated protein 2, chloroplastic) is also known as FBN1b or FIB1b (derived from Fibrillin-1b) and MYB90. Standardizing these names would facilitate research and communication.

Another research gap is the lack of a comprehensive summary of known regulatory proteins (transcription factors) that directly control specific genes and their transcription. A detailed list of proteins driving the expression of Chalcone synthase, the initial enzyme in the phenylpropanoid biosynthetic pathway, along with the promoter motifs ensuring interaction with other proteins, would greatly aid in understanding the complex regulation of these genes. Such a database, also including other genes, could clarify observed effects and discrepancies in gene expression and inspire new hypotheses regarding the regulation of protective metabolite production.

Furthermore, summarizing current knowledge on epigenetic modifications, such as DNA modifications, histone modifications, and non-B-DNA structures from multiple sources (sequencing and in silico predictions) would be valuable. Testing whether these modifications can be introduced into a given gene and assessing their impact on gene expression in the lab could provide significant insights.

Despite substantial progress in understanding transcriptional control of gene expression, our knowledge remains limited. Future studies should aim to unify nomenclature and test more complex hypotheses about gene expression coordination.

## 8. Conclusions

In this review, we combined current knowledge focused on the plant photoreceptors and related signaling pathways with the knowledge about the plant transcription factors driving the expression of genes related to the production of phenolic compounds. We are convinced that this thorough summarization of the current literature on this topic is very useful and can be used as inspiration for the design of new studies aimed at a comprehensive understanding of the signal mediated by different intensities and spectral composition of PAR and UV (and temperature) on PheCS production and profile. In addition to this, we pointed in the previous section (Section 7) to the current challenges and gaps in this field, together with the possible applications of this knowledge.

## Data Availability

No new data were created or analyzed in this study. Data sharing is not applicable to this article.
